# Influence of pulse duration and water/air cooling ratio on the efficiency of Er:YAG 2940 nm laser in debonding of porcelain laminate veneers: An in vitro study

**DOI:** 10.1002/cre2.554

**Published:** 2022-04-29

**Authors:** Mohand AlBalkhi, Omar Hamadah

**Affiliations:** ^1^ Department of Prosthodontics, Faculty of Dentistry Alsham Private University Damascus Syria; ^2^ Laboratory of Medical Lasers, The Higher Institute for Laser Research and Applications Damascus University Damascus Syria; ^3^ Oral Medicine Department, Faculty of Dentistry Damascus University Damascus Syria

**Keywords:** debonding, Er:YAG laser, PLV, pulse duration

## Abstract

**Objective:**

To determine the effectiveness of different pulse durations (PD) and the water/air (W/A) cooling ratio of the Er:YAG 2940 nm laser that are required for debonding porcelain laminate veneers (PLV), by investigation of the needed time for PLV debonding (DT) and the changes in dental pulp temperature.

**Materials and Methods:**

Thirty‐six extracted noncarious human maxillary premolars were prepared for receiving PLV. Samples were randomly assigned to six different groups, based on PD and the W/A ratio: Groups A (50 µs, 1:1), B (50 µs, 3:3), C (100 µs, 1:1), D (100 µs, 3:3), E (300 µs, 1:1), and F (300 µs, 3:3). Veneers were debonded using laser irradiation by the same parameters (270 mJ, 15 Hz) with noncontact application mode.

**Results:**

All 36 veneers were debonded. Samples of the 50 and 100 µs PDs showed significantly shorter DT (7.4−17 s) than that of the 300 µs which showed significantly the longest DT (104 s) among all other groups (*p* < .001). However, the highest elevation of pulp temperature was observed in Group E (300 µs, 1:1) which reached (3.4°C).

**Conclusion:**

Using the 50 or 100 µs PD of the Er:YAG laser was more efficient than 300 µs in reducing DT of PLVs with minimal change in pulp temperature. W/A cooling ratio had minimal influence on the DT of PLV.

## 1 INTRODUCTION

The porcelain laminate veneer (PLV), fabricated using ceramic materials, is a less invasive alternative to full coverage restorations, and is used for functional and cosmetic correction of many conditions; they have been shown to demonstrate good clinical performance and excellent bonding to the tooth structure (Sexana et al., 2020).

Removal of failed PLVs is a time‐consuming procedure generally performed with a rotary instrument and may require the removal of a considerable amount of underlying sound tooth structure (Anusavice, 2003). This is indicative of the significant strength of micromechanical bonding, between the fitting surface of PLV and prepared enamel of tooth by adhesive and luting resin cement (Chun et al., 2010; Stacey, 1993). Recently, the removal process has become much easier with the development of a PLV debonding technique, using an Er:YAG laser (Erbium‐doped: yttrium aluminum garnet) at a wavelength of 2940 nm, and Er,Cr:YSGG laser (erbium, chromium‐doped yttrium, scandium, gallium and garnet laser) at a wavelength of 2780 nm (Bader & Krejci, 2006; Olivi & Olivi, 2015). These lasers have been successfully used for debonding orthodontic ceramic brackets, owing to their high photonic absorption in water molecules, residual monomers, and adhesive bonding cement containing water that is also present in the PLV bonding layer (Azzeh & Feldon, 2003; Oztoprak et al., 2010; Tocchio et al., 1993).

The emitted laser beam penetrates the full thickness of PLV and causes thermal softening of the adhesive resin layer (Olivi and Olivi, 2015). This technique is still not well described in the scientific literature, and experimental in‐vitro studies in this field are insufficient. This is mainly due to difficulties related to the application of removal force on the well‐adapted cervical margin of PLV, which should be applied during or directly after laser application, just before the cooling down of the targeted adhesive resin layer (Iseri et al., 2014).

Very little data have been published in this field with different veneer removal techniques. Morford et al. (2011) investigated the efficiency of the Er:YAG laser in debonding PLVs using one technique with many different laser parameters; Iseri et al (2014) concluded that the application of the Er:YAG laser decreased the bond strength of laminate veneers; Oztoprak et al. (2012) used different application times of the Er:YAG laser and concluded that they were effective in debonding ceramic laminate veneers by softening the adhesive resin; Goznel et al. (2019) investigated the effects of different Er:YAG laser application parameters on shear bond strength values of all‐ceramic restorations cemented to different tooth surfaces, and they concluded that as the depth of prepared tooth structure increases, minimal values of laser parameters such as frequency and energy are needed for debonding of the same restoration type. With regard to temperature considerations, Alikhasi et al. (2019) used the Er,Cr:YSGG laser to debond different types of ceramic PLVs and concluded that there is no significant difference for debonding time among them without increasing pulpal temperature over 1°C. AlBalkhi et al. (2017) used different application modes of Er:YAG laser with various parameters of laser energy and frequency to get the best choices of parameters to be used for debonding of PLV without elevation of dental pulp temperature over a threshold of 5.5°C, considered as critical and irritating for dental pulp (Zach & Cohen, 1965). Other laser parameters like pulse durations (PD) and water/air (W/A) cooling ratio have not been studied yet.

The aim of this study was to determine the efficiency of Er:YAG laser for debonding PLVs without damaging the underlying sound tooth structure, by using different parameters of PD and W/A cooling ratio; associated areas of investigation were the time required to effect debonding and the changes in the degree of dental pulp temperature.

## 2 MATERIALS AND METHODS

**Figure 1:**
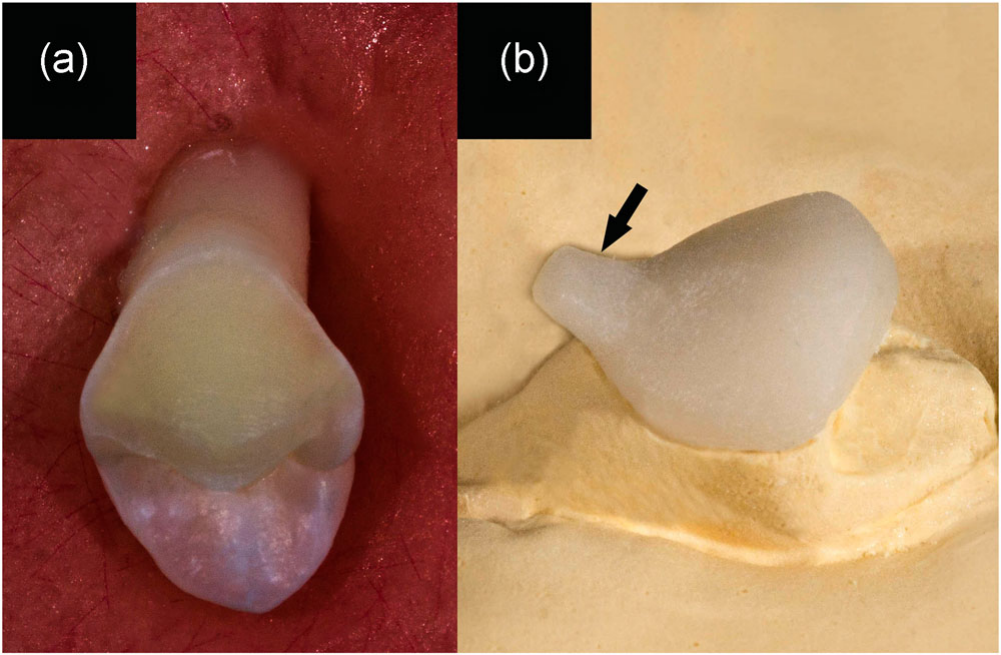
(a) Prepared premolar placed obliquely within acrylic resin mold. (b) PLV fabricated on a cast with a specially designed cervical prominence (arrowed). PLV, porcelain laminate veneers

Forty‐six extracted, noncarious human maxillary premolars stored in 0.5% chloramine T. solution were used. The teeth were placed obliquely in acrylic resin molds as their roots and lingual part of the crowns were covered within the resin (Figure 1a). Facial aspects of teeth were prepared within the enamel, for receiving PLVs in standardized dimensions (5 × 7 mm, 0.7 mm depth, and 0.5 mm at the cervical chamfer finishing line). The impressions were taken using polyvinyl siloxane (Elite HD; Zhermack, Italy) and sent to the laboratory for veneer fabrication. Lithium Disilicate IPS e.max LT ingots (Ivoclar, Vivadent, Schaan, Liechtenstein) were assigned for fabrication of PLVs with a thickness of 0.7 ± 0.05 mm according to the manufacturer's instructions. For experimental purposes, a 1 × 1 mm cervical prominence at a 45° angle was added to the veneer surfaces to hook the needed weight of the test (Figure 1b).

The cementation process of all PLVs was achieved according to standardized protocols. Hydrofluoric acid 10% was used for etching, applied for 20 s, and cleaned thoroughly with running water, followed by a silane film applied to veneer surfaces and left for 60 s. Simultaneously, the prepared surface of each tooth was etched using phosphoric acid 37% for 30 s, then washed and dried for equivalent periods. A light‐cured bonding agent (Tetric N‐Bond Universal, Ivoclar, Vivadent) was applied on etched surfaces and remained without curing. Subsequently, each veneer was cemented using light‐cured resin cement (Variolink N, Ivoclar, Vivadent, Schaan, Liechtenstein) and light‐cured for 60 s. All cemented samples were stored in distilled water at 37°C for 24 h.

**Figure 2:**
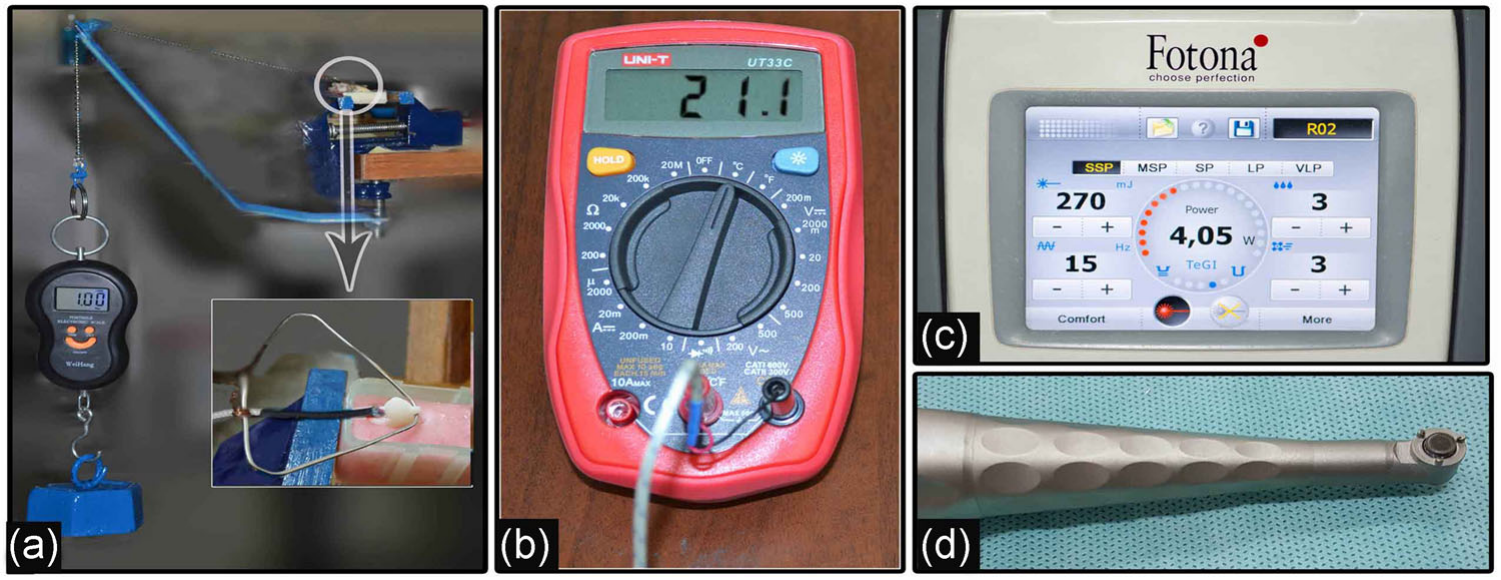
(a) A dislodging force applied on the cervical margin of PLV during laser application. (b) Multimeter with K‐type thermocouple. (c) Screen of Er:YAG laser device. (d) Noncontact laser handpiece. PLV, porcelain laminate veneers

Before laser application, a dislodging force of 1 kg was applied at a 45° angle applied at the cervical margin of the veneer, producing a 15‐N force (Figure 2a). A probe of thermocouple type K which connected to a digital multimeter (UNI‐T UT33C) was then attached to a pulp chamber of the sample to measure the changes in intra‐pulpal temperature during laser application (Figure 2b).

Ten samples were allocated for a pilot study purposed to assign the effective parameters of PD and W/A cooling ratio, to be used later in the main groups of this study for debonding of PLVs within a period of 150 s of continuous laser application. Laser irradiation was performed using the Er:YAG laser 2940 nm (Fotona‐Lightwalker; Slovenia, 2011) at an energy 270 mJ per pulse, and a frequency of 15 Hz (average power 4.05 W), using a noncontact application mode (7−8 mm distance) and Er:YAG laser handpiece (R02) (Figure 2c,d). Laser irradiation was applied perpendicular to the surface of each PLV and completed using a horizontal scanning motion.

The pulse duration parameters of the laser device were super short pulse (SSP = 50 µs), medium‐short pulse (MSP = 100 µs), short pulse (SP = 300 µs), long pulse (LP = 600 µs), and very long pulse (VLP = 1000 µs); each pulse width value was applied on two samples with different W/A cooling ratios of 1:1 and 3:3. Even after laser application for a period of 150 s, debonding of PLVs did not succeed when the PD was LP or VLP, whether the W/A cooling ratio was either 1:1 or 3:3. In consequence, those two PD parameters (LP and VLP) were excluded from the main study.

Thirty‐six samples then were randomly assigned to six different groups (A, B, C, D, E, and F) based on the two studied factors: PDs (SSP, MSP, and SP) and W/A cooling ratio (1:1 and 3:3). Each group comprised six samples.

All porcelain veneers of the examined groups were completely and easily removed using the Er:YAG laser, using the aforementioned parameters. The results were entered into the statistical program SPSS (V.22). In addition to standard descriptive statistical calculations (mean, standard deviation, median), the one‐way analysis of variance test was used in the comparison between the study groups, followed by the post‐hoc Tukey HSD test that was utilized to identify the differences; statistical significance was established at *p* < .05.

**Figure 3:**
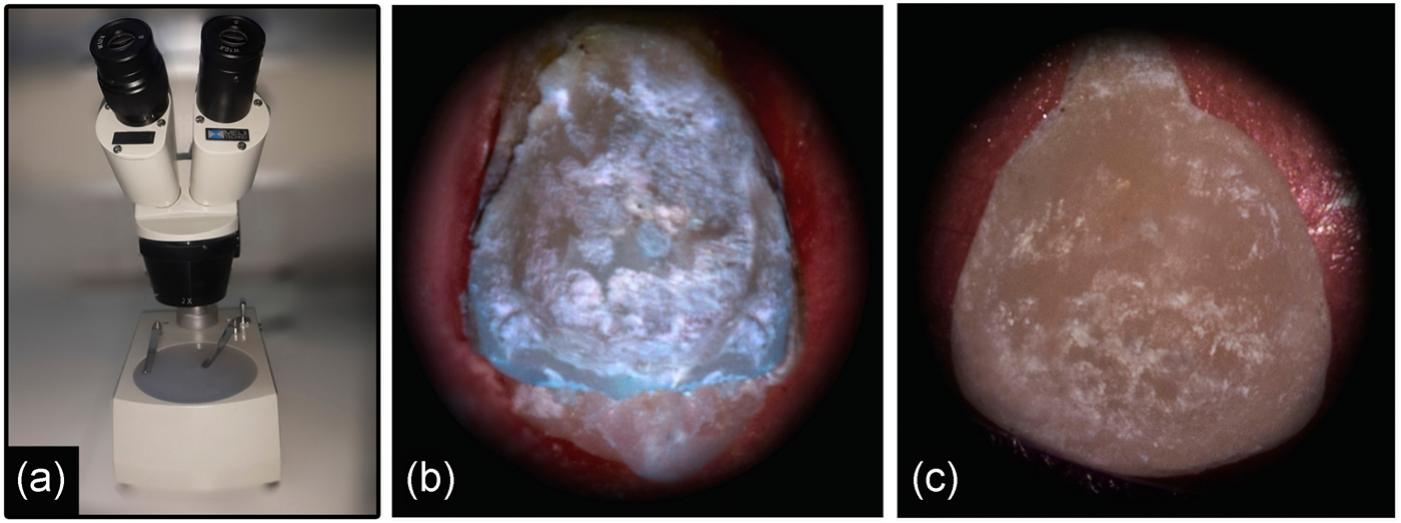
(a) A stereoscope adjusted at ×20 magnification. (b) Tooth surface after debonding. (c) Veneer surface after debonding

The fit surface of the debonded veneer and associated prepared surface of the tooth were examined stereoscopically at a ×20 magnification (stereoscope 1999; MEIJI Techno, Japan), to evaluate whether the failure modes occurred adhesively in the veneer/cement (V/C) interface, or in the enamel/cement (E/C) interface, or cohesively within the resin cement layer (Mak et al., 2002) (Figure 3).

## 3 RESULTS

Based on the results of the aforementioned pilot study, PLVs were successfully debonded when the pulse duration was SSP, MSP, and SP, but when the pulse duration was LP and VLP (whether W/A cooling ratio was either 1:1 or 3:3), debonding did not succeed until 150 s of laser application.

The mean values of debonding time (s) and temperature change (°C), and the respective standard deviation and standard error values are shown in Table 1.

**Table 1:** Descriptive statistics of study groups

	Group	*N*	Mean	Std. deviation	Std. error
Debonding time (s)					
	B	6	15.9	1.7	0.7
	C	6	11.8	1.0	0.4
	D	6	17.0	1.4	0.5
	E	6	29.3	3.5	1.4
	F	6	104.6	11.8	4.8
Temperature (°C)	A	6	1.2	0.2	0.09
	B	6	.6	0.3	0.13
	C	6	2.7	0.3	0.14
	D	6	1.3	0.2	0.11
	E	6	3.4	0.6	0.26
	F	6	1.7	0.3	0.15

Results of the GLM test revealed a significant difference between groups for each tested factor (debonding time and temperature change) with *p* < .001 (Table 2). Shapiro−Wilk normality test is used with each test and showed that the data are distributed normally (for temperature *p* = .06405 > .05 and time *p* = .8724).

**Table 2:** Results of ANOVAtest for study groups

	ANOVA				
	Sum of squares	df	Mean square	*F*	Sig.
Time					
Pulse duration	18.312	2	9.156	769.92	<0.0001
W/A ratio	5.762	1	5.762	484.50	<0.0001
Pulse duration × W/A ratio	1.248	2	0.624	52.47	<0.0001
Residuals	0.357	30	0.012		
Temperature					
Pulse duration	0.12435	2	0.06218	60.63	<0.0001
W/A ratio	0.09243	1	0.09243	90.131	<0.0001
Pulse duration × W/A Ratio	0.00851	2	0.00425	4.149	0.025
Residuals	0.03076	30	0.00103		

Time and temperature were affected by PD and W/A cooling ratio and the interaction between them (*p* < .05, Table 2).

Tukey HSD was used to see how much the time and temperature were affected by the interaction between the PD and the W/A cooling. Short PD (SP, 300 µs) with (3:3) W/A cooling ratio (Group F) showed significantly longest debonding time (104 s) among all the study groups. However, the highest elevation of pulp temperature was observed in Group E (300 µs, 1:1) which reached (3.4°C), but it still did not exceed a threshold of 5.25°C (Table 3).

**Table 3:** Tukey HSD multiple comparison test of averages of debonding time (s) and averages of temperature change (°C)

	Tukey HSD debonding time (s)		Tukey HSD temperature (°C)	
Group	Difference	*p* value	Difference	*p* value
C−A	0.471	<.001	0.127	<.001
E−A	1.376	<.001	0.176	<.001
B−A	0.765	<.001	−0.058	.039
D−A	0.833	<.001	0.009	.997
F−A	2.648	<.001	0.048	.136
E−C	0.905	<.001	0.049	.115
B−C	0.294	<.001	−0.185	<.001
D−C	0.363	<.001	−0.118	<.001
F−C	2.178	<.001	−0.079	<.001
B−E	−0.611	<.001	−0.234	<.001
D−E	−0.543	<.001	−0.167	<.001
F−E	1.273	<.001	−0.128	<.001
D−B	0.068	.884	0.067	.012
F−B	1.883	<.001	0.106	<.001
F−D	1.815	<.001	0.039	.316

Samples of all groups showed a similar adhesive failure mode in the enamel/cement interface, where most of the cement remained on the surface of the tooth and only very small remnants of cement were detected on veneer surfaces (Figure 3b,c).

## 4 DISCUSSION

Published study data relating to the debonding of PLVs using erbium laser are still few and not well covered when compared with those that focus on the debonding of orthodontic ceramic brackets (Azzeh & Feldon, 2003; Ma et al., 1997; Nalbantgil et al., 2014; Oztoprak et al., 2010; Tocchio et al., 1993). This may in part be due to difficulty in the application of debonding force on a very thin margin of veneer during laser application. As used previously in the article published in 2017 (AlBalkhi et al., 2017), we designed a method to apply a dislodging force on the cervical margin of the veneer in a way similar to what happens clinically, to test the debonding process in a laboratory setting; this has greater similarity to clinical reality than the application of shear forces by Instron device to test shear bond strength, as seen in most of the previous studies designed for debonding porcelain veneer or orthodontic ceramic brackets (Oztoprak et al., 2010; Tocchio et al., 1993). Using a specially designed cervical prominence of the veneer (1 × 1) at a 45°angle, a loading force of 15 N was applied. The 15‐N force was selected to be the median among measured root scaling force values, which was determined previously from 8 to 23 N (Villanueva et al., 2007). In order for the force to be applied, a 1 kg weight should be hung by a chain with a ring hooked onto the cervical prominence of the veneer at an angle of 45°. The equivalent weight determined is based on the following equation [Weight = Force × cos45/acceleration of gravity (9.8)] which results in 1.08 kg.

Access to the pulp chamber was carried out just before the time of laser application to avoid pulp tissue dehydration, which may affect the measuring process of pulp temperature (Nalbantgil et al., 2014; Walinski et al., 2021).

Similar parameters of laser energy, frequency, and application mode were gained from outcomes of our previously published research, performed using similar methods (AlBalkhi et al., 2017).

This study was designed as a complement to the previous study published in 2017 (AlBalkhi et al., 2017), which aimed to test two variables that had not been studied before (PD and W/A cooling ratio), to establish an ideal criterion for using the Er:YAG laser in debonding PLVs. The parameters were selected according to the results of a pilot study, which was performed on 10 samples. The pilot study was conducted by applying the five PD parameters provided by the Er:YAG laser device (Fotona‐Lightwalker; Slovenia, 2011): (SSP = 50 µs), (MSP = 100 µs), (SP = 300 µs), (LP = 600 µs), (VLP = 1000 µs), with either a 1:1 or 3:3 W/A cooling ratio. The effective parameters were selected depending on their effectiveness in veneer debonding within a 150‐s period of continuous laser application.

The influence of W/A cooling ratio on dental pulp temperature was more significant when longer PD (SP, 300 µs) was used; it is suggested that with this pulse duration, a greater pulpal cooling period between pulses may be achieved when compared with shorter PD (SSP, 50 µs) and (MSP, 100 µs).

All selected parameters succeeded in debonding PLVs with different periods, which is in agreement with the studies of Goznel et al. (2019), AlBalkhi et al. (2017), Iseri et al. (2014) Oztoprak et al. (2012), and Morford et al. (2011), in investigating the efficiency of Er:YAG laser in debonding PLVs using different debonding techniques.

The study of failure mode is an indicative factor of the laser penetration's depth within layers of PLV, resin cement, and tooth surface, and provides a valuable indicator of possible enamel damage. Most samples showed that resin cement had remained on the tooth surface, so the failure was within the veneer/cement interface; this indicated that there is no discernable risk of tooth damage. This result conforms with studies by Morford et al. (2011) and AlBalkhi et al. (2017), which stated that the majority of failures occurred in the veneer/cement interface and surfaces of veneers that were free of cement. The similarity between groups in failure mode, in spite of different PD and W/A cooling ratios used, may demonstrate a minimal influence of these applied parameters on the debonding process, compared with other laser parameters such as energy and frequency as presented in the study of AlBalkhi et al. (2017).

## 5 CONCLUSION

Within the limitations of this study, it was concluded that both SSP duration (50 µs) and MSP duration (100 µs) were efficient, fast, and safe parameters of Er:YAG laser for the removal of lithium disilicate PLV, when using the same laser energy and frequency parameters of noncontact application mode, and irrespective of a W/A cooling ratio of 1:1 or 3:3.

The W/A cooling ratio had minimal influence on the debonding time and pulp temperature when shorter PD were used (SSP, 50 µs, MSP, 100 µs). All selected parameters in this study did not raise the dental pulp temperature beyond a threshold of 5.5°C, throughout the time duration of PLV removal using Er:YAG laser irradiation.

## CONFLICTS OF INTEREST

The authors declare no conflicts of interest.

## ETHICS STATEMENT

This study was approved by the Damascus University Ethical Committee under decision no. 163 WD. We certify that the study was performed in accordance with the ethical standards as laid down in the 1964 Declaration of Helsinki and its later amendments or comparable ethical standards. This article does not contain any studies involving animals performed by any of the authors.

## INFORMED CONSENT

A consent form was obtained initially from all patients who underwent tooth extraction and provided those teeth that were used in this study.

## AUTHOR CONTRIBUTIONS

Mohand AlBalkhi designed the study, collected sample, methodology (prepared samples for the test), collected data (measurements) then entered them into the statistical software (SPSS) and made analysis, wrote most parts of the manuscript, and edited according to the *CEDR* journal. Omar Hamadah shared in the design of the study, methodology (applied laser on samples), wrote an interpretation of the statistical analysis, revised the manuscript, and edited the manuscript.

## Data Availability

The data sets used and/or analyzed during the current study are available from the corresponding author on reasonable request.
